# Functional Analysis of HSD17B3-Deficient Male Mice Reveals Roles for HSD17B7 and HSD17B12 in Testosterone Biosynthesis

**DOI:** 10.1210/endocr/bqaf078

**Published:** 2025-05-08

**Authors:** Ben M Lawrence, Liza O’Donnell, Anne-Louise Gannon, David A Skerrett-Byrne, Shanmathi Parameswaran, Imogen Abbott, Sarah Smith, David J Handelsman, Diane Rebourcet, Lee B Smith

**Affiliations:** College of Engineering, Science and Environment, The University of Newcastle, Callaghan, New South Wales 2308, Australia; School of BioSciences, The University of Melbourne, Parkville, Victoria 3010, Australia; Office of the Deputy Vice-Chancellor (Research), Griffith University, Southport, Queensland 4222, Australia; Hudson Institute of Medical Research and Department of Molecular and Translational Sciences, School of Clinical Sciences, Monash University, Clayton, Victoria 3168, Australia; College of Engineering, Science and Environment, The University of Newcastle, Callaghan, New South Wales 2308, Australia; Infertility and Reproduction Research Program, Hunter Medical Research Institute, New Lambton, New South Wales 2305, Australia; College of Engineering, Science and Environment, The University of Newcastle, Callaghan, New South Wales 2308, Australia; Infertility and Reproduction Research Program, Hunter Medical Research Institute, New Lambton, New South Wales 2305, Australia; College of Health, Medicine and Wellbeing, The University of Newcastle, Callaghan, New South Wales 2308, Australia; Institute of Experimental Genetics, Helmholtz Zentrum München, German Research Center for Environmental Health, Neuherberg 85764, Germany; German Center for Diabetes Research (DZD), Neuherberg 85764, Germany; College of Engineering, Science and Environment, The University of Newcastle, Callaghan, New South Wales 2308, Australia; College of Engineering, Science and Environment, The University of Newcastle, Callaghan, New South Wales 2308, Australia; College of Engineering, Science and Environment, The University of Newcastle, Callaghan, New South Wales 2308, Australia; University of Sydney and Andrology Department, ANZAC Research Institute, Concord Hospital, Sydney, New South Wales 2139, Australia; College of Engineering, Science and Environment, The University of Newcastle, Callaghan, New South Wales 2308, Australia; Irset (Institut de recherche en santé, environnement et travail)-UMR_S 1085, Univ Rennes, Inserm, EHESP, Rennes 35065, France; Office of the Deputy Vice-Chancellor (Research), Griffith University, Southport, Queensland 4222, Australia

**Keywords:** testosterone, male fertility, androgens, testis, hydroxysteroid dehydrogenase

## Abstract

Historically, 17β-hydroxysteroid dehydrogenase type 3 (HSD17B3) was thought to be the key enzyme responsible for testicular testosterone production. In humans, loss-of-function mutations in *HSD17B3* impair testosterone production during prenatal life leading to impaired development of androgen-dependent tissues in 46,XY individuals. However, male mice with HSD17B3 deficiency exhibit normal testicular testosterone concentrations, normal development of reproductive organs and are fertile, suggesting that mice express other hydroxysteroid dehydrogenase enzymes capable of testicular testosterone synthesis. This study aimed to investigate whether 17β-hydroxysteroid dehydrogenase type 12 (HSD17B12), which can convert androstenedione to testosterone in mice but not in humans, compensates for the lack of HSD17B3 in *Hsd17b3* knockout (KO) mice. We used CRISPR/Cas9 to substitute the amino acid in mouse HSD17B12 that is responsible for its ability to convert androstenedione to testosterone with the amino acid of the human enzyme that prevents androstenedione being used as a substrate. When this *Hsd17b12* mutation was introduced into *Hsd17b3* KO mice, males exhibited normal reproductive tracts but reduced testicular testosterone production with a consequential reduction in seminal vesicle weight. This suggests HSD17B12 contributes toward testosterone production in the absence of HSD17B3, but other enzymes must also contribute. We therefore quantified other testicular hydroxysteroid dehydrogenases, finding that HSD17B7 mRNA and protein was markedly upregulated in *Hsd17b3* KO testes. We confirmed that mouse, but not human, HSD17B7 can produce testosterone in vitro. We conclude that compared to humans, mice exhibit increased plasticity in testosterone production via hydroxysteroid dehydrogenase enzymes to support androgen action and male fertility.

The production and action of the androgens testosterone and dihydrotestosterone (DHT) are essential for male sexual development and function, including fertility (1, 2). Hydroxysteroid 17β dehydrogenases convert 17-ketosteroids to 17β-hydroxysteroids, and 17β-hydroxysteroid dehydrogenase type 3 (HSD17B3), expressed in humans solely in the testis, is the primary enzyme responsible for the conversion of androstenedione to testosterone in the Leydig cells of the testis, culminating in testosterone secretion. Loss-of-function mutations to the human *HSD17B3* gene results in HSD17B3 deficiency whereby individuals with XY chromosomes exhibit androgen-deficient (hypogonadal) external genitalia but masculinized internal Wolffian structures at birth (1, 3-5). In contrast, *Hsd17b3* knockout (KO) mice develop with normal testosterone production and male reproductive function, including fertility when mature (6, 7). In *Hsd17b3* KO mice, testosterone levels remain much higher in the testes than in circulation and other peripheral tissues including the adrenal glands, epididymis, prostate, and white adipose tissue, suggesting that the continued testosterone production in these mice originates from the testis (6, 7). Although the adrenal glands contribute a small proportion to androgen biosynthesis in human males (8), rodent adrenals do not produce androgens due to a lack of CYP17A1 enzyme expression (9, 10). Since steroidogenic enzyme expression in the mouse adrenal glands can change following orchidectomy (11), a previous study investigated whether the adrenal glands could contribute to testosterone production in *Hsd17b3* KO mice (7). Adrenalectomized *Hsd17b3* KO mice did not result in a decrease in circulating androstenedione, testosterone or DHT (7). These findings strongly suggest that the continued testosterone production in the testes of adult *Hsd17b3* KO mice is due to the expression of alternative testosterone synthesizing enzymes in the testis. However, the identity of these enzyme(s) is unknown.

Other hydroxysteroid dehydrogenase enzymes can catalyze the conversion of androstenedione to testosterone. HSD17B5 (also known as AKR1C3) can perform this conversion (12, 13) but is undetectable in the mouse testis of both wild-type and *Hsd17b3* KO adult mice (6, 14). HSD17B1 is expressed in fetal mouse Sertoli cells and contributes to testosterone production during fetal development (15, 16) and it can compensate for a lack of HSD17B3 during fetal development (17). However, in adulthood, *Hsd17b1* is undetectable in the testis in wild-type and *Hsd17b3* KO mice and cannot compensate for *Hsd17b3* deficiency (6, 17). Therefore, HSD17B1 and HSD17B5 are unlikely to contribute to testicular testosterone production in adult *Hsd17b3* KO mice.

HSD17B12 can convert androstenedione to testosterone in mice but not in humans (18), and produces 11-ketotestosterone, the predominant androgen in some fish (19). *Hsd17b12* encodes an enzyme that converts the estrogenic precursor estrone to the biologically active estrogen, estradiol (20), and catalyzes reactions involved in fatty acid elongation (21, 22) which is essential for early embryonic development (21, 23, 24). HSD17B12 was suggested as a potential candidate to compensate for a lack of HSD17B3 in *Hsd17b3* KO mice because it is expressed by adult Leydig cells (6).

Important functional differences exist between mouse and human HSD17B12. The differences in substrate specificity between the mouse and human enzymes regarding testosterone production is due to a key amino acid at position 234 on the amino acid sequence (18). In human and primate HSD17B12, a bulky phenylalanine amino acid at this location prevents the entrance of C19-steroids into the active site of the enzyme (20). In contrast, in mouse HSD17B12, the leucine amino acid at position 234 is smaller, and allows C19-steroids to enter the active site of the enzyme more readily than in humans (18). These data suggest that the inability of human HSD17B12 to make testosterone means it cannot compensate for the absence of HSD17B3 in conditions of human HSD17B3 deficiency. However, it is possible that HSD17B12 could maintain testosterone biosynthesis in the absence of *Hsd17b3* in mice.

The current study aimed to investigate the mechanisms by which testosterone production continues in adult *Hsd17b3* KO mice, with a focus on HSD17B12. As the loss of *Hsd17b12* is embryonically lethal in mice (21, 23), we used CRISPR/Cas9 to produce a novel mouse model in which we substituted the leucine amino acid in mouse HSD17B12 that is responsible for its ability to produce testosterone from androstenedione with phenylalanine, the amino acid in the human enzyme that prevents androstenedione being used as a substrate. We cross-bred this mutation into a homozygous *Hsd17b3* KO line to determine whether mouse HSD17B12 contributes to testosterone production in *Hsd17b3* KO mouse testes.

## Materials and Methods

### Transgenic Mice

Transgenic mice were generated at the MEGA Genome Engineering Facility at the Garvan Institute of Medical Research, Darlinghurst, NSW. CRISPR/Cas9 was used to generate a 7-base pair deletion at the end of exon 1 of *Hsd17b3* resulting in a frameshift mutation. These mice exhibited a phenotype indistinguishable from our previously generated *Hsd17b3* KO line (6, 14).

CRISPR/Cas9 was also used to generate the mutated *Hsd17b12* transgenic mouse line. Homologous repair using a template containing the desired mutation in the DNA sequence altered the 234th amino acid in the mouse HSD17B12 protein sequence. At this location, the DNA coding sequence for a leucine amino acid was altered to code for a phenylalanine amino acid to mimic the human HSD17B12 (referred as L234F).

A male C57BL/6 mouse carrying the homozygous KO of *Hsd17b3* was mated to a female C57BL/6 mouse carrying the homozygous L234F mutation to generate a double transgenic mouse line. The breeding strategy used thereafter to test the impact of the mutated HSD17B12 protein in *Hsd17b3* KO mice involved mating mice that were homozygous *Hsd17b3* KO and heterozygous *Hsd17b12* mutation.

Mice were exposed to a 12-hour day/night cycle and had access to water and soy-free chow, ad libitum. All procedures were approved by The University of Newcastle's Animal Care and Ethics Committee (ACEC), approval number #A-2018-820. All animal experiments were performed in accordance with the Australian code of practice for the care and use of animals for scientific purposes by the National Health and Medical Research Council of Australia.

### In Vivo Treatments

Mice were injected intraperitoneally with a hyper-stimulating dose (20 IU) of human chorionic gonadotropin (hCG; Sigma-Aldrich, Australia) 16 hours prior to tissue collection (6).

### Tissue Collection

Day-80 adult mice were euthanized by CO_2_ inhalation. Blood was collected via cardiac puncture, centrifuged at 10 000*g* for 10 minutes at 4 °C, and serum was then collected and snap-frozen. Mice were weighed and anogenital distance (AGD) was measured using digital calipers (Adelab Scientific, Thebarton, SA, Australia). Androgen-dependent tissues were excised and weighed. Serum and tissues were either snap-frozen and stored at −80 °C until used for later analysis or fixed in Bouin's solution for 6 hours for histological analysis.

### Genotyping

Genotyping was performed on ear biopsies after weaning and confirmation of genotype was performed again postmortem on tail tissue. Genomic DNA (gDNA) was digested in Tris-EDTA-Tween, pH 8, and Proteinase K for 1 hour at 55 °C, and then for 7 minutes at 95 °C to denature remaining Proteinase K. The digested samples were diluted 1:10 in DEPC-treated DNase- and RNase-free sterile water. The genotype of transgenic mice was identified by transgene-specific polymerase chain reaction (PCR) assays. PCR was performed on gDNA extracts using a Type-it Mutation Detect PCR Kit (QIAGEN, VIC, Australia). Details of primers used are shown in Tables 1 and 2. PCR products were detected using a QIAxcel DNA high resolution kit on the QIAxcel Advanced System and analyzed by QIAxcel ScreenGel Software (QIAGEN, VIC, Australia). *Il2* primers were included when genotyping *Hsd17b12* as a positive control for the PCR.

**Table 1.** Details of genotyping assays

Assay	Reagent	Volume per reaction (μL)	Annealing temperature (°C)	Product size (bp)
*Hsd17b3*	Type-it Mastermix (2×)	5	55	WT-150
	*Hsd17b3* forward primer (20 μM)	0.1		
	*Hsd17b3* WT-reverse primer (20 μM)	0.1		Heterozygous-144 and 150
	*Hsd17b3* del-reverse primer (20 μM)	0.1		
	dH_2_0	3.7		Homozygous-144
	gDNA	1		
*Hsd17b12* WT	Type-it Mastermix (2×)	5	63	*Hsd17b12* WT-436
	*Hsd17b12* WT-forward primer (20 μM)	0.1		
	*Hsd17b12* WT-reverse primer (20 μM)	0.1		
	*Il2* forward primer (20 μM)	0.1		*Il2* Control-324
	*Il2* reverse primer (20 μM)	0.1		
	dH_2_0	3.6		
	gDNA	1		
*Hsd17b12* Mutant	Type-it Mastermix (2×)	5	63	*Hsd17b12* Mut-432
	*Hsd17b12* mut-forward primer (20 μM)	0.1		
	*Hsd17b12* mut-reverse primer (20 μM)	0.1		
	*Il2* forward primer (20 μM)	0.1		*Il2* Control-324
	*Il2* reverse primer (20 μM)	0.1		
	dH_2_0	3.6		
	gDNA	1		

**Table 2.** Primers used for genotyping assays

Gene	Forward primer(s)	Reverse primer(s)
*Hsd17b3*	*Hsd17b3* forward: ggagaagctcttcattgctg	*Hsd17b3* WT-reverse: cttacctgcccattgtcccat
		*Hsd17b3* del-reverse: cttacctgtcccattgatcg
*Hsd17b12*	*Hsd17b12* WT-forward: acattagaaacctcacgctgct	*Hsd17b12* WT-reverse: ttgccagttttgtagctacaagg
	*Hsd17b12* mut-forward: acattagaaacctcacgctgct	*Hsd17b12* Mut-forward: cagttttgtcgcgacgaa
*Interleukin-2* (*Il2*) (Positive control gene)	*Il2* forward: ctaggccacagaattgaaagatct	*Il2* reverse: gtaggtggaaattctagcatcatcc

### Transformation and Purification of Plasmids

Plasmids were transformed into NEB^®^ Stable Competent *E. coli* (C3040I) cells following the NEB high efficiency transformation protocol (New England BioLabs, Notting Hill, VIC, Australia). In brief, 50 to 100 ng of plasmid DNA was added to NEB^®^ Stable Competent *E. coli* and incubated on ice for 30 minutes, before being heat shocked at 42 °C for 30 seconds, then placed back on ice for 5 minutes. NEB^®^ 10-beta/Stable outgrowth medium (New England BioLabs, Notting Hill, VIC, Australia) was added to each sample followed by incubation at 30 °C for 60 minutes, with constant shaking at 250 rpm. Cells were then spread over ampicillin-selective agar plates and incubated for 16 hours at 37 °C.

Transformed bacterial colonies were grown and purified following the QIAGEN Plasmid Maxi Kit Purification Handbook. Following purification of plasmid DNA, air-dried pellets were resuspended in DEPC-treated DNase- and RNase-free sterile water (Thermo Fisher Scientific, Vic, Australia). The yield of plasmid DNA was measured using the NanoDrop Lite Spectrophotometer (Thermo Fisher Scientific, VIC, Australia). Plasmids were stored at −30 °C.

### Site-Directed Mutagenesis

Mouse *Hsd17b12* plasmid (details in Table 3) was altered by inducing a mutation that substituted the leucine amino acid at position 234 to a phenylalanine amino acid. The mutation and plasmid transformation were performed using an Agilent QuikChange II XL Site-Directed Mutagenesis Kit (Integrated Sciences, Chatswood, NSW, Australia). Primers were designed as recommended to induce a mutation to the mouse *Hsd17b12* (forward primer- gcagagtgtcatgccata**ctt**cgtagctacaaaactggc; reverse primer- gccagttttgtagctacg**aag**tatggcatgacactctgc, with bold and underlined regions indicating the point mutations) (Sigma-Aldrich, Australia). Manufacturer positive control and samples were prepared following manufacturer guidelines and cycled on a MasterCycler X50s. Amplified parental (nonmutated) products were digested with 10 U of *Dpn I* restriction enzyme at 37 °C for 1 hour. *Dpn I*-treated DNA was then added to XL10-GOLD ultracompetent cells and incubated on ice for 30 minutes, followed by heat-shock at 42 °C for 30 seconds, then placed back on ice to transform the cells with plasmid DNA. Cells were grown briefly in NZY^+^ broth at 37 °C for 1 hour before being spread onto LB-ampicillin-selective agar plates containing 5-bromo-4-chloro-3-indolyl-β-D-galactopyranoside (X-gal) and isopropyl β-D-1-thiogalactopyranoside (IPTG). Plates were incubated for 16 hours at 37 °C and single colonies containing the mutated plasmid were grown. The mutated plasmid was grown and purified following the QIAGEN Plasmid Maxi Kit Purification Handbook.

**Table 3.** Details of plasmids used to transfect HEK-293T cells

Plasmid	Developer	Product ID	Tag/Reporter	Antibiotic resistance
e*GFP* control	GeneCopoeia	EX-NEG-M61	N/A	Ampicillin
Mouse *Hsd17b3*	GeneCopoeia	EX-Mm33978-M61	IRES2-eGFP	Ampicillin
Mouse *Hsd17b12*	GeneCopoeia	EX-Mm07555-M61	IRES2-eGFP	Ampicillin
Human *HSD17B12*	GeneCopoeia	EX-V1191-M61	IRES2-eGFP	Ampicillin
Mouse *Hsd17b7* (variant 2)	GeneCopoeia	EX-Mm30153-M61	IRES2-eGFP	Ampicillin
Human *HSD17B7* (variant 1)	Vector Builder	pDNA(VB220915-1259dkg)	eGFP	Ampicillin

### DNA Sequencing

**Figure 1.**
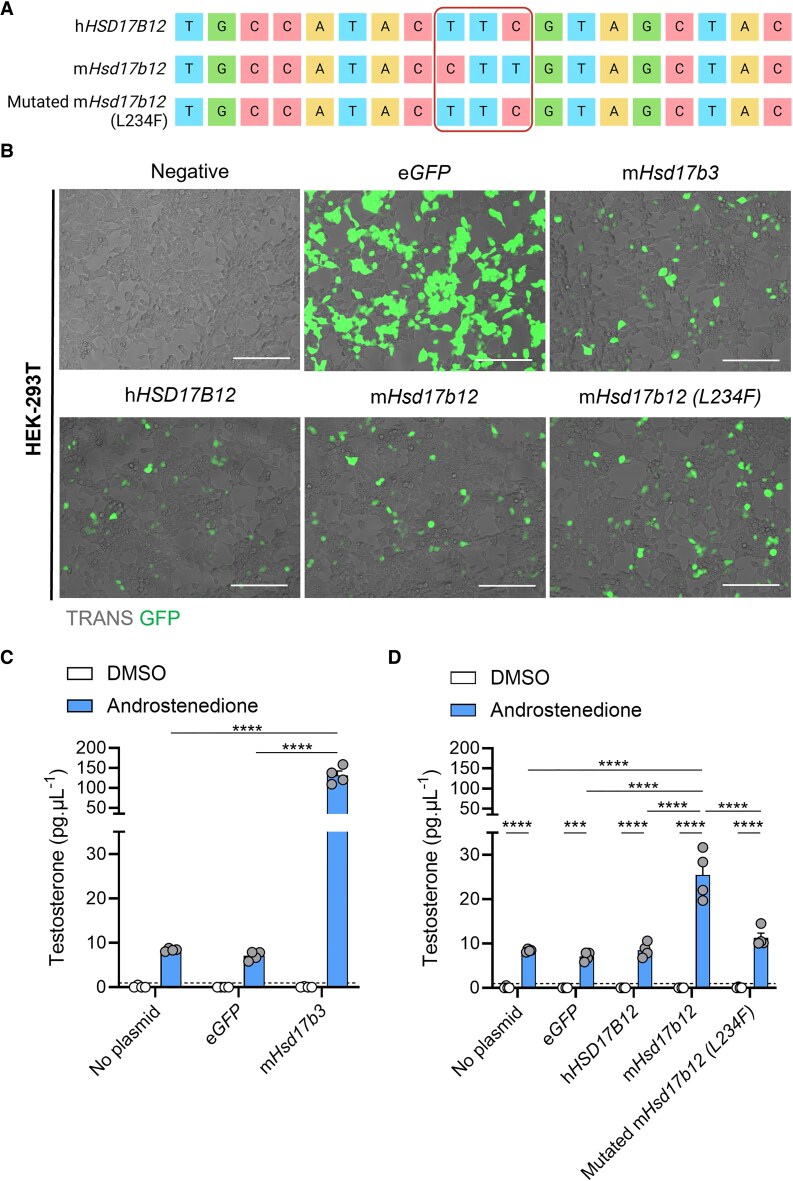
The presence of a leucine residue at amino acid position 234 in mouse HSD17B12 enables the conversion of androstenedione to testosterone. (A) Site-directed mutagenesis was performed on plasmids containing the genetic sequence of mouse (m)*Hsd17b12* to alter a single leucine amino acid to a phenylalanine (L234F). Plasmids containing the genetic sequence for human (h) *HSD17B12*, m*Hsd17b12*, and mutated m*Hsd17b12* (L234F) were sequenced by Sanger sequencing. Created with BioRender.com. (B) HEK-293T cells were transfected with plasmids carrying the e*GFP* reporter gene and steroidogenic enzyme genes, and testosterone in the culture media was quantified by mass spectrometry. Cells were transfected and treated with either DMSO (vehicle, open bars) or 150 ng/mL androstenedione (blue bars) for 24 hours. Representative images of HEK-293T cells that were transfected with plasmids expressing eGFP (green) alone (eGFP), or plasmids expressing eGFP, and either m*Hsd17b3*, h*HSD17B12*, m*Hsd17b12*, or a mutated m*Hsd17b12* which had a leucine amino acid substituted with a phenylalanine amino acid (L234F). Scale bar: 150 μm. TRANS: transillumination (C) Testosterone produced by cells transfected with m*Hsd17b3* were used as a positive control for the conversion of androstenedione to testosterone. (D) Testosterone produced by cells transfected with wild-type m*Hsd17b12*, h*HSD17B12*, and m*Hsd17b12* carrying the L234F mutation. Testosterone limit of detection = 0.98 pg/mL and is denoted by the dotted black line on the *y*-axis. Technical triplicates were averaged and plotted as biological replicates (n = 4). Two-way ANOVA, Tukey's multiple comparisons test, data shown as mean ± SEM. Significant differences between groups are indicated as *** = *P* ≤ .001, **** = *P* ≤ .0001.

**Figure 2.**
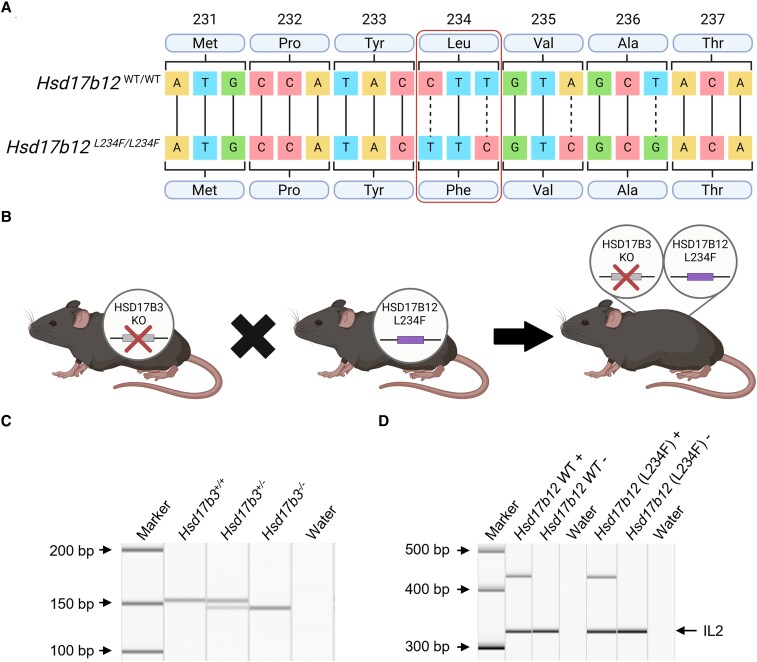
Generation and validation of *Hsd17b3* knockout (KO) mice expressing HSD17B12^L234F^, with a mutation in amino acid position 234 that prevents its ability to convert androstenedione to testosterone. (A) Sanger sequencing of mouse *Hsd17b12* in wild-type and mutated animals. Point mutations in the DNA sequence were made to change the leucine (Leu) amino acid to a phenylalanine (Phe) amino acid, altering the amino acid sequence to replicate that seen in the human protein sequence. The mutated sequence is referred to as L234F. Created with BioRender.com. (B) Mutated *Hsd17b12* mice (*Hsd17b12*^L234F^) were cross-bred with *Hsd17b3* KO mice to generate a mouse line incorporating both the *Hsd17b3* null allele and the *Hsd17b12* mutation. Created with BioRender.com. (C, D) Genotypes of mice were determined using standard PCR. (C) Presence of the *Hsd17b3*^+/+^ (wild-type) allele was indicated by a 153-base pair (bp) band. The *Hsd17b3*^−/−^ (*Hsd17b3* KO) allele was indicated by a 7-bp deletion which showed a 146-bp band. (D) The presence or absence (+ or −) of the *Hsd17b12* wild-type gene and the mutated *Hsd17b12* (L234F) gene was identified by PCR products that were 436 and 432 bp, respectively. Interleukin-2 (IL-2) was used as a positive control in amplified samples, shown by a 324-bp band.

The successful mutation of a leucine to a phenylalanine amino acid at site 234 in the *Hsd17b12* plasmid and in the humanized mouse line was confirmed by Sanger sequencing by the Australian Genome Research Facility (Westmead, Sydney, Australia). The desired point mutation was confirmed (Figs. 1A, 2A). Two separate point mutations were also induced in the mouse line; however, neither of these altered the amino acid sequence (Fig. 2A).

### Cell Culture

HEK-293T cells were cultured in DMEM/F12 (Thermo Fisher Scientific, VIC, Australia) supplemented with 10% FCS (CellSera Australia, Rutherford, NSW, Australia) and 1% penicillin-streptomycin (Sigma-Aldrich, Australia). Cells received fresh culture medium approximately every 2 to 3 days and were incubated at 37 °C with 5% CO_2_.

### Transfections and Cell Treatments

HEK-293T cells (ATCC, Manassas, Virginia, USA) were transfected with plasmids to test the function of genes (Table 3), using a Lipofectamine™ 3000 transfection reagent kit (Thermo Fisher Scientific, VIC, Australia) as per manufacturer guidelines. One day prior, cells were seeded at 65% confluency in a 96-well plate, and 30 minutes prior to transfection, cells were washed and medium was replaced with Opti-MEM™ medium (Thermo Fisher Scientific, VIC, Australia) supplemented with 1% penicillin-streptomycin (Sigma-Aldrich, Australia), and incubated at 37 °C with 5% CO_2_. A mastermix containing 0.1 μg plasmid DNA, 0.15 μL Lipofectamine™ 3000 reagent, 0.2 μL P3000™ Reagent (2 μL/μg DNA), and 10 μL Opti-MEM™ medium, per well was added to each well. Culture plates were placed on an orbital shaker for 5 minutes at 100 rpm to ensure plasmids were spread evenly across cells, followed by incubating at 37 °C with 5% CO_2_. Transfection was confirmed after 24 hours by the presence of enhanced green fluorescent protein (eGFP) using the EVOS M5000 Imaging System (Thermo Fisher Scientific, Vic, Australia).

After transfection, cells were washed and medium replaced with phenol red free DMEM/F12, supplemented with 10% FCS and 1% penicillin-streptomycin. At 72 hours post transfection, cells were treated with either diluted dimethylsulfoxide (DMSO; Sigma-Aldrich, Australia) as a vehicle control or with 150 ng/mL of androstenedione (Cayman Chemical Company, Ann Arbor, MI, USA). Androstenedione was initially prepared as a 10 mg/mL stock concentration in DMSO. Stock androstenedione was then diluted in complete phenol red free DMEM/F12 medium. DMSO vehicle controls were diluted by the same factor. Each treatment group was performed in triplicate. Treated cells were incubated for 24 hours at 37 °C with 5% CO_2_ followed by media being collected from cells and stored at −80 °C until steroid analysis.

### Histology

Testis and epididymis tissues fixed in Bouin's solution were processed and embedded in paraffin wax. Then 5-μm sections were prepared, dewaxed in xylene (Sigma-Aldrich, Australia), and rehydrated through a decreasing gradient of ethanol. Tissue sections were stained with hematoxylin and eosin (Sigma-Aldrich, Australia). Light microscopy images of tissues were captured using a Zeiss AXIO Imager.A2 microscope (Carl Zeiss AG, Germany) with Olympus cellSens imaging software (Olympus, Macquarie Park, NSW, Australia).

### Quantitative Reverse Transcriptase PCR

RNA was extracted from whole testis tissue using the RNeasy Mini kit (QIAGEN, VIC, Australia) as per manufacturer's instructions, incorporating RNase-free DNase on-column digestion (QIAGEN, VIC, Australia). An external Luciferase RNA control (Promega, Alexandria, NSW, Australia) was added at 1 ng/20 mg tissue to the homogenized tissue during the RNA extraction. RNA concentration was measured using a NanoDrop Lite Spectrophotometer (Thermo Fisher Scientific, VIC, Australia). Extracted RNA was reverse transcribed to cDNA using the SuperScipt VILO cDNA Synthesis Kit (Thermo Fisher Scientific, VIC, Australia) as per manufacturer instructions. Water (no template) and reverse transcriptase negative (-RT) controls were included in all reverse transcriptions. For quantitative reverse transcriptase (qRT)-PCR, target-specific primers and corresponding specific probes were identified and selected using the online Roche Universal Probe Library (UPL) Assay Design Centre. The qRT-PCR was performed on the LightCycler 96 system (Millenium Science, Mulgrave, VIC, Australia). Primers used to assess mouse *Hsd17b7* include: forward- gctcactgtgacaccgtacaa, and reverse- ctccggtttttggtggaag, along with UPL probe 81. Luciferase external control was detected using the primers: forward- gcacatatcgaggtgaacatcac, and reverse- gccaaccgaacggacattt, and a TaqMan Probe (tacgcggaatacttc). Quantification of mRNA expression was calculated using the 2^−ΔΔCt^ method. Gene expression was determined relative to the external housekeeping Luciferase gene in adult tissues (Roche, AU). Each sample was run in triplicate and an average of the Ct value was taken.

### Measurement of Testosterone in Cell Culture Media

Testosterone produced in vitro was quantified by liquid chromatography–mass spectrometry (LC-MS) using a QTRAP^®^ 6500 LC-MS/MS System (SCIEX, Toronto, Canada) by the Central Analytical Facility at The University of Newcastle. Testosterone (Cayman Chemical Company, Ann Arbor, MI, USA) used for standards was resuspended in DMSO (Sigma-Aldrich, Australia) to make a stock concentration of 1 mg/mL. A testosterone working concentration of 1000 pg/μL was made using stock testosterone and fresh culture media (DMEM/F12 + 10% FBS). A 1:2 serial dilution was performed and used to make a standard curve with the lowest standard being 0.98 pg/mL.

### Circulating and Intratesticular Steroid Analysis

Circulating and intratesticular steroids were quantified by LC-MS. Fragments of adult mouse testes (20-40 mg) were homogenized in 50 mM Tris pH 7.4, 0.01% SDS, 1% deoxycholate, containing cOmplete Mini Protease Inhibitor Cocktail (Sigma-Aldrich, Australia) and PhosSTOP (Sigma-Aldrich, Australia) at a concentration of 20 μL/mg of tissue. Samples were homogenized using a TissueLyser II (QIAGEN, VIC, Australia) for 4 × 30-second intervals at 25 Hz. Samples were placed on ice for 1 minute after each 30-second interval to avoid samples overheating. LC-MS analysis was performed at the ANZAC Research Institute, Concord Hospital, NSW.

### Proteomics

#### Sample preparation

Proteomics was performed on fragments of whole testes from adult (day 80) wild-type and *Hsd17b3* KO mice that were generated in a previous study (14). The *Hsd17b3* KO mice used for proteomics were also heterozygous for a null allele of the *Srd5a1* gene (14); however, these mice showed an identical endocrine and reproductive phenotype as previously generated *Hsd17b3* KO mouse lines (6, 7, 14). Testis fragments were homogenized as described above in ice cold lysis buffer containing 0.1M Na_3_CO_3_ pH 11 (Sigma-Aldrich, Australia), 10mM Na_3_VO_4_ (Sigma-Aldrich, Australia), 2.5% Protease inhibitor cocktail (Sigma-Aldrich, Australia), and one tablet of PhosSTOP (Sigma-Aldrich, Australia) per 10 mL (25-27). Samples were sonicated for 4 × 10-second intervals, with samples incubated on ice for 20 seconds between each interval to prevent protein denaturation. Total protein was quantified by the Pierce^TM^ BCA Protein Assay kit (Thermo Fisher Scientific, Vic, Australia) and 500 μg of protein from each sample was mixed 1:1 with urea/thiourea to reach a final concentration of 6M urea/2M thiourea. Proteins were reduced by the addition of dithiothreitol (DTT) (Sigma-Aldrich, Australia) to a final concentration of 10 mM and incubated at room temperature for 30 minutes, shaking at 400 rpm. Proteins were then alkylated by adding iodoacetamide (Sigma-Aldrich, Australia) to a final concentration of 20mM and incubated in the dark at room temperature for 30 minutes with 400 rpm shaking. Protein samples were subsequently digested with a 1:12.875 ratio of 1 μg/μL trypsin/Lys-C Mix (Promega, Alexandria, NSW, Australia), incubated at room temperature for 3 hours with shaking at 400 rpm. Urea concentration was diluted to below 1M using 50mM of triethylammonium bicarbonate (TEAB) buffer (Sigma-Aldrich, Australia), pH 7.8, and incubated at 37 °C for 16 hours with shaking at 1000 rpm. Lipids were precipitated by adding formic acid (Sigma-Aldrich, Australia) to make a final concentration of 2%, and then centrifuged at 14 000*g* for 10 minutes at room temperature. The supernatant containing the peptides was stored at −80 °C until samples were desalted.

Peptide samples were cleaned and desalted using commercial desalting columns (Oasis, Waters Corporation, Milford MA, USA). Clean peptides were eluted through the equilibrated desalting columns using a vacuum pump. Peptides were quantified using a Qubit 2.0 fluorometer (Thermo Fisher Scientific, Vic, Australia) and 10 μg of sample peptides were lyophilized using a RVC 2-25 SpeedyVac (Martin Christ, Gefriertrocknungsanlagen GmbH, Germany) set at 45 °C for 2.5 hours. Lyophilized peptides were stored at −80 °C until required for mass spectrometry analysis.

#### Nano-liquid chromatography–mass spectrometry

Lyophilized clean peptides were resuspended in 2% acetonitrile (Thermo Fisher Scientific, VIC, Australia), 0.1% TFA to a final concentration of 1 μg/μL. Reverse phase nano-LC-MS was performed using an Orbitrap Eclipse Tribrid MS equipped with a front-end field asymmetric ion mobility spectrometry (FAIMS), coupled to a Vanquish Neo ultra high-performance liquid chromatography system UHPLC System (Thermo Fisher Scientific, Waltham, MA, US). Samples were loaded onto an Acclaim PepMap 100 C18 75 μm × 20 mm trap column (Thermo Fisher Scientific, Vic, Australia) for pre-concentration and online de-salting. Separation was then achieved using an EASY-Spray PepMap C18 75 μm × 250 mm column (Thermo Fisher Scientific, Vic, Australia), employing a linear gradient of acetonitrile (2%-40%) over 85 minutes. Full MS/data dependent acquisition MS/MS mode was utilized on Xcalibur (Thermo Fisher Scientific; version 4.6.67.17) with an automatic cycle between 3 compensations voltages (CV; −50, −65, and −80). The Orbitrap mass analyzer was set at a resolution of 1 200 000, to acquire full MS with an m/z range of 375-1500, with a normalized automatic gain control target set to standard and maximum fill times set to auto. The 20 most intense multiply charged precursors were selected for higher-energy collision dissociation fragmentation with a collisional energy of 35%. MS/MS fragments were measured using the Ion Trap, with the scan rate set to rapid, using standard mode for automatic gain control target and automatic maximum fill times.

#### Proteomic data processing and analysis

As per previous proteomic studies (25, 28, 29), database searching of raw files were performed using Proteome Discoverer 2.5 (Thermo Fisher Scientific), utilizing SEQUEST HT to search against the UniProt Mus musculus database (25 444 sequences, downloaded November 29, 2022). Database searching parameters included up to 2 missed cleavages, a precursor mass tolerance set to 10 ppm and fragment mass tolerance of 0.02 Da. Trypsin was designated as the digestion enzyme. Cysteine carbamidomethylation was set as a fixed modification while acetylation (K, N-terminus) and oxidation (M) were designated as dynamic modifications. Interrogation of the corresponding reversed database was also performed to evaluate the false discovery rate (FDR) of peptide identification using Percolator based on q-values, which were estimated from the target-decoy search approach. To filter out target peptide spectrum matches over the decoy-peptide spectrum matches, a fixed FDR of 1% was set at the peptide level.

### Statistical Analysis

Statistical analyses were performed using GraphPad Prism software, versions 8.4.3 and 10.2.3 (GraphPad Software, San Diego, CA, USA). Gaussian distribution was assessed by the Shapiro-Wilk normality test to determine if downstream analyses would be performed using appropriate parametric or nonparametric statistical testing. Datasets which passed normality testing underwent parametric statistical tests including unpaired *t* tests, one-way ANOVA with Tukey's post hoc test, or two-way ANOVA with Tukey's post hoc test. Datasets which did not pass the normality test underwent nonparametric statistical testing including Kruskal-Wallis test with Dunn's post hoc test.

Significance testing and comparisons associated with the proteomic analysis of testis tissue were calculated by Proteome Discoverer 2.5 using a non-nested pairwise ratio approach, whereby the program calculates the peptide group ratios as the geometric median of all combinations of ratios from all the replicates for each group. Protein ratios were subsequently calculated as the geometric median of the peptide group ratios and statistical testing was completed using a Student's *t* test. Proteomic data was considered significantly different when the *P* value ≤.05.

## Results

### Replacement of Leucine With Phenylalanine at Position 234 Inhibits Testosterone Biosynthesis in Mouse HSD17B12

We first aimed to confirm that mouse HSD17B12, but not human HSD17B12, can use androstenedione as a substrate to produce testosterone, and this is due to the reduced size of the amino acid at position 234.

Substitution of the leucine (Leu) amino acid with a phenylalanine (Phe) amino acid at position 234 (L234F) was confirmed through Sanger sequencing (Fig. 1A). The *Hsd17b12* sequences in both the mouse and human *Hsd17b12* plasmids matched exactly to previously published data (18). To assess the ability of mouse and human HSD17B12 to synthesize testosterone, we transfected HEK-293T cells with plasmids expressing GFP reporters and steroidogenic enzymes (Fig. 1B). Non-transfected cells and eGFP controls produced low levels of testosterone upon addition of androstenedione, suggesting some endogenous hydroxysteroid dehydrogenase activity in HEK-293T cells (Fig. 1C and 1D). Transfection with mouse *Hsd17b3* induced a >15-fold increase in testosterone compared to controls (Fig. 1C), indicating that these cells can be used to assess 17β-hydroxysteroid dehydrogenase activity.

The inability of human HSD17B12 to utilize androstenedione as a substrate (18) was confirmed by the unchanged amount of testosterone produced by cells transfected with human *HSD17B12* compared to controls (Fig. 1D). In contrast, transfection of cells with mouse *Hsd17b12* caused a significant increase in testosterone production (Fig. 1D), confirming species-dependent 17β-hydroxysteroid dehydrogenase activity.

Introduction of the L234F mutation into mouse *Hsd17b12* transfected cells significantly reduced its ability to produce testosterone compared to the nonmutated mouse *Hsd17b12* transfected cells (Fig. 1D). Testosterone concentrations in androstenedione-treated cells transfected with the mutated mouse *Hsd17b12* were not different to cells transfected with human *HSD17B12* (Fig. 1D). These results confirm the functional differences between the mouse and human HSD17B12 enzymes in their ability to convert androstenedione to testosterone (18) and demonstrate that the ability of mouse HSD17B12 to produce testosterone can be inhibited by the introduction of a phenylalanine amino acid at residue 234.

### The Generation of *Hsd17b3*-Deficient Mice Expressing a Mutated *Hsd17b12*

We hypothesized that the maintenance of masculinization and fertility in *Hsd17b3* KO male mice could be due to the ability of endogenous HSD17B12 to maintain testicular testosterone concentrations. Complete deletion of *Hsd17b12* in mice is embryonically lethal (23) so CRISPR/Cas9 technology was used to generate a mouse line in which *Hsd17b12* was mutated from a leucine amino acid to a phenylalanine amino acid to mimic the human HSD17B12 (denoted *Hsd17b12^L234F^* mice). The L234F mutation was confirmed by Sanger sequencing (Fig. 2A). This mutation prevents the ability to convert androstenedione to testosterone (Fig. 1D) but is unlikely to prevent HSD17B12's ability to convert estrone to estradiol (18). If HSD17B12 is the sole enzyme producing testosterone in *Hsd17b3*-deficient adult mice, then the introduction of this mutation should cause a phenotype of androgen insufficiency in adult males.

*Hsd17b12^L234F^* mice were cross-bred with *Hsd17b3* KO mice to generate a mouse line incorporating both mutations (Fig. 2A and 2B). Genotyping confirmed mice were homozygous for the *Hsd17b3* KO (*Hsd17b3*^−/−^) by a single band that contained a 7-bp deletion (146 bp) compared to wild-type (WT; *Hsd17b3*^+/+^) and heterozygous (*Hsd17b3*^+/−^) which had a single 153 bp band or 2 bands, respectively (Fig. 2C). Separate PCRs were used to assess the presence or absence of the *Hsd17b12* WT and mutated genes for each mouse (Fig. 2D). The genotype of mice with a KO of *Hsd17b3* and expressing a mutated *Hsd17b12* with a L234F amino acid substitution is referred to as *Hsd17b3*^−/−^; *Hsd17b12*^L234F/L234F^.

### Male *Hsd17b3*-Deficient Mice Expressing Mutated *Hsd17b12* Have Smaller Seminal Vesicles but Remain Fertile

**Figure 3.**
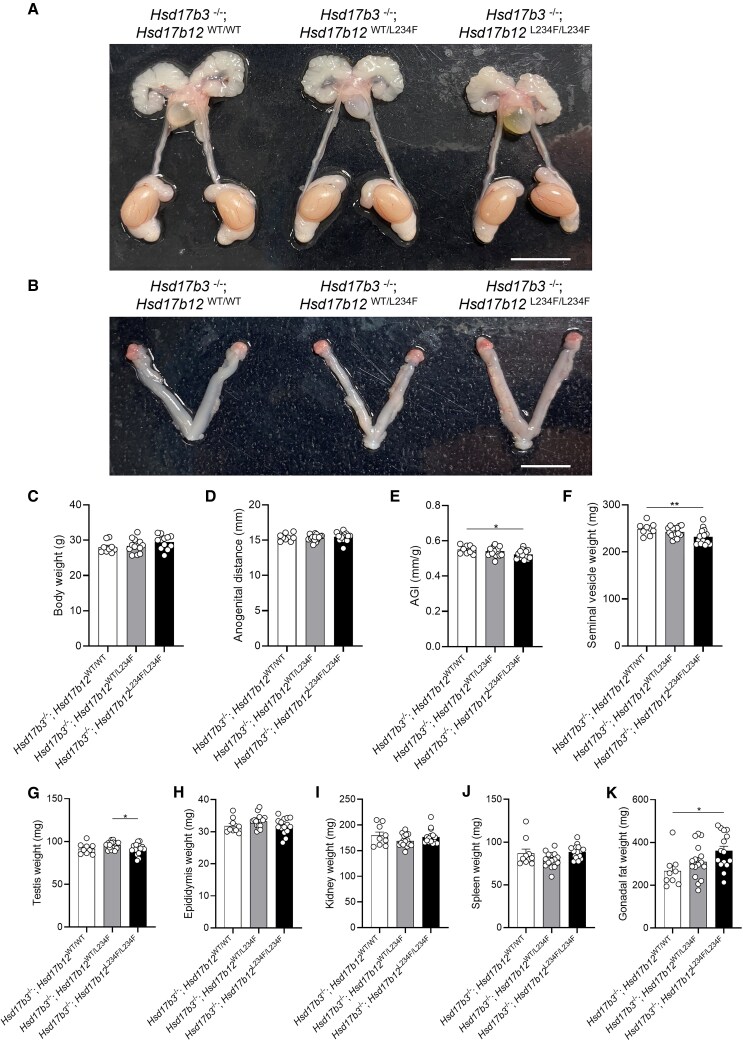
Characterization of the reproductive tract in adult (day 80) male *Hsd17b3* knockout (*Hsd17b3*^−/−^) mice expressing the mutant *Hsd17b12*^L234F/L234F^ allele. (A) Representative images of male and (B) female reproductive tracts of *Hsd17b3*^−/−^; *Hsd17b12*^WT/WT^, *Hsd17b3*^−/−^; *Hsd17b12*^WT/L234F^ and *Hsd17b3*^−/−^; *Hsd17b12*^L234F/L234F^ mice. Scale bars: 10 mm. (C) Total body weight, (D) anogenital distance, (E) anogenital index (AGI) (standardization of anogenital distance relative to body weight), (F) seminal vesicle, (G) testis, (H) epididymis, (I) kidney, (J) spleen, and (K) gonadal fat weights of male mice. One-way ANOVA, Tukey's test where *P* ≤ .05, data shown as mean ± SEM with n = 9-16. Significant differences between groups are indicated as * = *P* ≤ .05, ** = *P* ≤ .01.

The impact of the *Hsd17b12* L234F mutation on the phenotype of *Hsd17b3* KO male mice was characterized in adult (day 80) mice. Both males and females were examined to investigate whether a phenotype of under-virilization or disordered sexual development was evident (Fig. 3). All male reproductive tissues were present, including the testes, epididymides, vas deferens, seminal vesicles and prostate (Fig. 3A). All females had ovaries and uteri present in all genotypes (Fig. 3B). These results indicate that androgen bioactivity in male *Hsd17b3* KO mice expressing a mutated *Hsd17b12* continues during development. No change in body weight was observed between *Hsd17b3*^−/−^; *Hsd17b12*^WT/WT^, *Hsd17b3*^−/−^; *Hsd17b12*^WT/L234F^ and *Hsd17b3*^−/−^; *Hsd17b12^L234F/L234F^* groups (Fig. 3C). While there was no change in the anogenital distance (AGD) between any group (Fig. 3D), there was a decrease in the anogenital index (AGI), which is a standardized assessment of AGD (30), in *Hsd17b3*^−/−^; *Hsd17b12^L234F/L234F^* compared to *Hsd17b3*^−/−^; *Hsd17b12*^WT/WT^ (Fig. 3E). There was a significant reduction in seminal vesicle weight in *Hsd17b3*^−/−^; *Hsd17b12^L234F/L234F^* mice compared to *Hsd17b3*^−/−^; *Hsd17b12*^WT/WT^ controls (Fig. 3F), which is known to be sensitive to reductions in androgens (31). There was a slight but significant reduction in testis weight of *Hsd17b3*^−/−^; L234F*^L234F/L234F^* compared to *Hsd17b3*^−/−^; L234F^WT/L234F^ mice, but not compared to *Hsd17b3*^−/−^; L234F^WT/WT^ controls (Fig. 3G). No significant differences were detected in the weight of the epididymis (Fig. 3H), kidney (Fig. 3I), or spleen (Fig. 3J). There was also a significant increase in gonadal fat weight in *Hsd17b3*^−/−^; *Hsd17b12^L234F/L234F^* mice compared to the *Hsd17b3*^−/−^; *Hsd17b12*^WT/WT^ controls (Fig. 3K).

**Figure 4.**
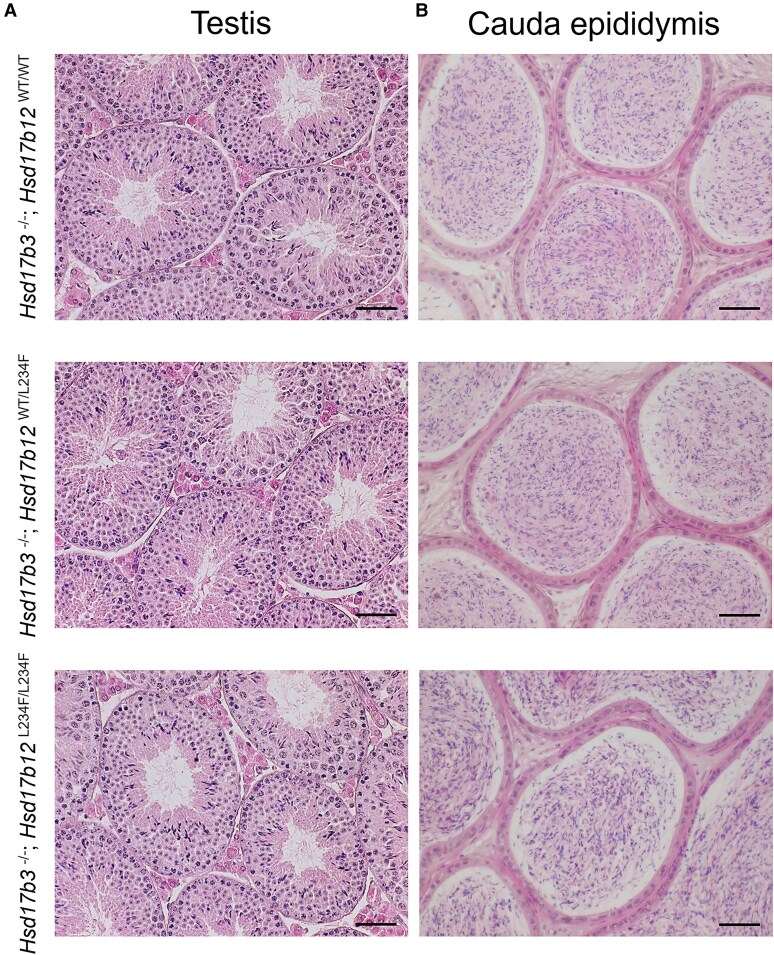
Testis and cauda epididymis histology in adult (day 80) male *Hsd17b3* knockout (*Hsd17b3*^−/−^) mice expressing either the mouse *Hsd17b12* wild-type allele (*Hsd17b12*^WT/WT^), heterozygous for the mutant allele (*Hsd17b12*^WT/L234F^) or homozygous for the humanized *Hsd17b12* mutant allele (*Hsd17b12*^L234F/L234F^). (A) Representative hematoxylin and eosin (H&E) staining of adult the testis and (B) cauda epididymis. Scale bars: 50 μm.

Gross testis histology was unchanged in *Hsd17b3*^−/−^; *Hsd17b12^L234F/L234F^* mice, with seminiferous tubules and interstitial cells resembling other genotypes (Fig. 4A) and the cauda epididymis contained abundant sperm (Fig. 4B). In a small number of test matings between *Hsd17b3*^−/−^; *Hsd17b12^L234F/L234F^* males and control females, live viable offspring were obtained.

### Testicular Testosterone Production Is Reduced in *Hsd17b3*-Deficient Mice Expressing Mutated *Hsd17b12*

We next investigated the impact of the L234F mutation in mouse HSD17B12 on testicular testosterone production in *Hsd17b3* KO mice in vivo. Adult mice were treated with hCG to stimulate maximal androgen production and intratesticular steroids were measured by LC-MS.

**Figure 5.**
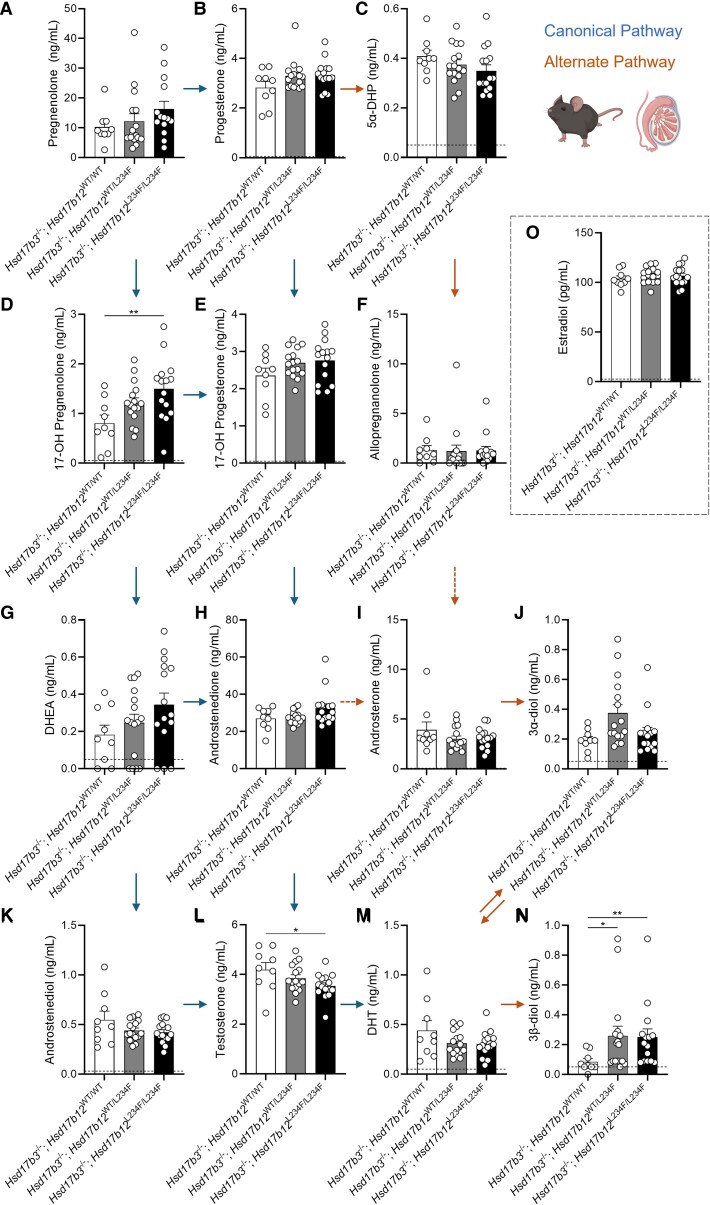
Intratesticular steroid concentrations in adult (day 80) male *Hsd17b3* knockout (*Hsd17b3*^−/−^) mice expressing the mutant *Hsd17b12*^L234F^ allele(s). Mice were treated with human chorionic gonadotrophin (hCG) to stimulate maximal steroidogenesis. Androgens and androgen precursors include (A) pregnenolone, (B) progesterone, (C) 5α-dihydroprogesterone (5α-DHP) (D) 17-OH pregnenolone, (E) 17-OH progesterone (F) allopregnanolone (G) dehydroepiandrosterone (DHEA), (H) androstenedione, (I) androsterone, (J) 5α-androstane-3α, 17β-diol (3α-diol), (K) androstenediol, (L) testosterone, (M) dihydrotestosterone (DHT), and (N) 5α-androstane-3β, 17β-diol (3β-diol). The arrows indicate the direction of the canonical and alternate androgen production pathways. Dotted arrows indicate an indirect conversion. Biological replicates that were below the limit of detection were recorded as 0 ng/mL. Limit of detection ranged from 0.01 ng/mL to 0.05 ng/mL depending on the analyte and is indicated by a dotted black line on *y*-axis. One-way ANOVA, Tukey's test (for parametric data) or Kruskal-Wallis test (for nonparametric data), where *P* ≤ .05, data shown as mean ± SEM with n = 9-16 per group. Significant differences between groups are indicated as * = *P* ≤ .05, ** = *P* ≤ .01. (O) Intratesticular estradiol levels. Limit of detection = 2.5 pg/mL and is indicated by the dotted black line on *y*-axis, One-way ANOVA, Tukey's test where *P* ≤ .05, data shown as mean ± SEM with individual values for n = 9-16 biological replicates per group.

Within the canonical pathway of androgen biosynthesis, pregnenolone, progesterone, 17-OH progesterone, dehydroepiandrosterone (DHEA), androstenedione, and androstenediol concentrations were unchanged in the testis (Fig. 5A, 5B, 5E, 5G, 5H, and 5K). Increased concentrations of 17-OH pregnenolone were observed in *Hsd17b3*^−/−^; *Hsd17b12^L234F/L234F^* compared to *Hsd17b3*^−/−^; *Hsd17b12*^WT/WT^ (Fig. 5D), pointing to other roles for the L234 residue in steroid metabolism. Importantly, *Hsd17b3* KO mice with the *Hsd17b12* L234F mutation showed a significant (∼16%) reduction in intratesticular testosterone concentrations compared to *Hsd17b3*^−/−^; *Hsd17b12*^WT/WT^ mice (Fig. 5L). These data suggest that HSD17B12 contributes to testosterone biosynthesis in the testes of *Hsd17b3* KO mice, but it is not solely responsible for the continued testosterone production. While there was a reduction in testosterone concentration, no changes in DHT were observed (Fig. 5M).

We also measured the impact of the L234F mutation in HSD17B12 on intratesticular androgen precursors within the alternate pathway of androgen biosynthesis, including 5α-dihydroprogesterone (5α-DHP), allopregnanolone, androsterone, 5α-androstane-3α,17β-diol (androstanediol [3α-diol]) and 5α-androstane-3β,17β-diol (3β-diol) (Fig. 5C, 5F, 5I, 5J, and 5N). No significant changes were seen in these steroids except for an increase in 3β-diol in both *Hsd17b3*^−/−^; *Hsd17b12^L234F/L234F^* and *Hsd17b3*^−/−^; *Hsd17b12*^WT/L234F^ testes (Fig. 5N), perhaps suggestive of alterations in 3β-diol production in mice with the mutated *Hsd17b12* allele.

Intratesticular concentrations of the estrogens estrone (E1) and estradiol (E2) were also quantified. While estrone was undetectable in all samples, no changes were observed in intratesticular estradiol between all genotypes (Fig. 5O).

Taken together, the results suggest that mouse HSD17B12 can contribute to testicular testosterone biosynthesis in *Hsd17b3* KO mice; however, it is not the sole enzyme responsible for the maintenance of adult testicular testosterone production in the absence of HSD17B3.

### Circulating Testosterone Concentrations Are Unchanged in *Hsd17b3*-Deficient Mice Expressing a Mutated *Hsd17b12*

**Figure 6.**
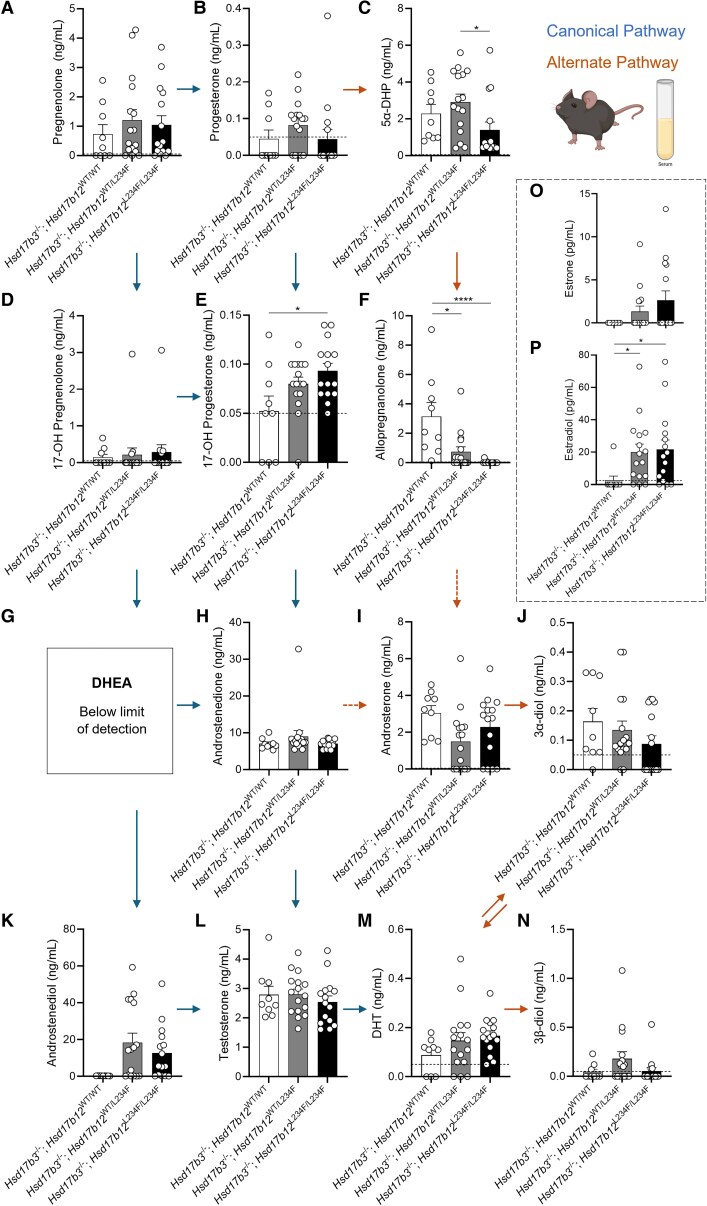
Circulating steroid concentrations in adult (day 80) male *Hsd17b3* knockout (*Hsd17b3*^−/−^) mice expressing the mutant *Hsd17b12*^L234F^ allele. Mice were treated with human chorionic gonadotrophin (hCG) to stimulate maximal steroidogenesis. Androgens and androgen precursors include (A) pregnenolone, (B) progesterone, (C) 5α-dihydroprogesterone (5α-DHP) (D) 17-OH pregnenolone, (E) 17-OH progesterone (F) allopregnanolone (G) dehydroepiandrosterone (DHEA), (H) androstenedione, (I) androsterone, (J) 5α-androstane-3α, 17β-diol (3α-diol), (K) androstenediol, (L) testosterone, (M) dihydrotestosterone (DHT), and (N) 5α-androstane-3β, 17β-diol (3β-diol). Steroids were quantified from the serum collected from mice. The arrows indicate the direction of the canonical and alternate androgen production pathways. Dotted arrows indicate an indirect conversion. Biological replicates that were below the limit of detection were recorded as 0 ng/mL. The limit of detection ranged from 0.01 ng/mL to 0.05 ng/mL depending on the analyte and is indicated by a dotted black line on *y*-axis. One-way ANOVA, Tukey's test (for parametric data) or Kruskal-Wallis test (for nonparametric data), where *P* ≤ .05, data shown as mean ± SEM with n = 9-16 per group. Significant differences between groups are indicated as * = *P* ≤ .05, **** = *P* ≤ .0001. (O) Quantification of estrone and (P) estradiol concentrations in serum. Limit of detection = 2.5 pg/mL and is indicated by the dotted black line on *y*-axis, One-way ANOVA, Kruskal-Wallis test where *P* ≤ .05, data shown as mean ± SEM with individual values for n = 9-16 biological replicates per group.

We also investigated circulating steroids in hCG-treated adult *Hsd17b3* KO male mice expressing the L234F mutation, as *Hsd17b12* is ubiquitously expressed (18). Within the canonical pathway of androgen biosynthesis, no significant changes were observed in pregnenolone, progesterone, 17-OH pregnenolone, androstenedione, or androstenediol (Fig. 6A, 6B, 6D, 6H, and 6K). 17-OH progesterone concentrations were significantly increased in *Hsd17b3*^−/−^; *Hsd17b12*^L234F/L234F^ compared to *Hsd17b3*^−/−^; *Hsd17b12*^WT/WT^ mice (Fig. 6E). Dehydroepiandrosterone (DHEA) was undetectable (Fig. 6G) suggesting that the Δ4 canonical pathway remains the preferred route for androgen biosynthesis in mice lacking *Hsd17b3.* Importantly, no differences were observed in circulating concentrations of testosterone or DHT among any of the genotypes (Fig. 6L and 6M).

In the alternate pathway of androgen biosynthesis, *Hsd17b3*^−/−^; *Hsd17b12*^L234F/L234F^ mice showed reduced concentrations of 5α-dehydroepiandrosterone (5α-DHP) in circulation compared to *Hsd17b3*^−/−^; *Hsd17b12*^WT/L234F^ mice (Fig. 6C). Both *Hsd17b3*^−/−^; *Hsd17b12*^WT/L234F^ and *Hsd17b3*^−/−^; *Hsd17b12*^L234F/L234F^ mice exhibited reduced concentrations of allopregnanolone compared to *Hsd17b3*^−/−^; *Hsd17b12*^WT/WT^ mice (Fig. 6F). Interestingly, *Hsd17b3*^−/−^; *Hsd17b12*^L234F/L234F^ mice showed a statistically significant reduction in circulating concentrations of allopregnanolone compared to controls (Fig. 6F), suggesting that the mutated HSD17B12 may impact on the biosynthesis of allopregnanolone in peripheral tissues, but not in the testis (Fig. 5F). No differences were observed in the other alternate androgen precursors, including androsterone, 3α-diol, and 3β-diol (Fig. 6I, 6J, and 6N).

Androstenedione and testosterone are aromatized to estrone and estradiol, respectively, and mouse HSD17B12 can convert estrone to the more potent estrogen, estradiol (18). No significant differences were observed in estrone concentrations among the groups; however, there is high variability in the data of the *Hsd17b3*^−/−^; *Hsd17b12*^WT/L234F^ and *Hsd17b3*^−/−^; *Hsd17b12*^L234F/L234F^ cohorts (Fig. 6O). Circulating estradiol concentrations were increased in *Hsd17b3* KO mice expressing either the heterozygous or homozygous L234F *Hsd17b12* allele compared to *Hsd17b3* KO mice expressing wild-type *Hsd17b12* (Fig. 6P); however, whether this is a direct result of altered enzyme activity in converting estrone to estradiol, or via another mechanism remains to be established.

### Identification of HSD17B7 as Another Mouse Hydroxysteroid Dehydrogenase Enzyme that Can Synthesize Testosterone

**Figure 7.**
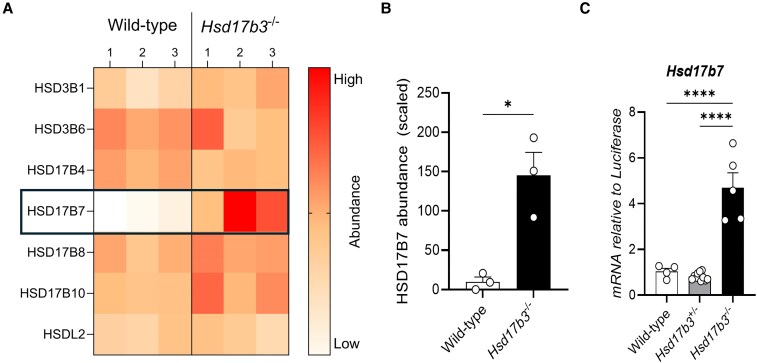
Upregulation of HSD17B7 protein and mRNA in HSD17B3-deficient mice. (A) Heat map representing the protein abundance of all hydroxysteroid dehydrogenases detected in wild-type and *Hsd17b3* knockout (KO; *Hsd17b3*^−/−^) mice. The color scale indicates protein abundance. (B) Scaled abundance values of HSD17B7 in wild-type and *Hsd17b3* KO mice. Data shown as scaled in relation to whole abundance of proteins detected. Scaling of data was performed on Proteome Discoverer 2.5 software for interpretation. Limit of detection on mass spectrometer was 0.2 (scaled). Biological replicates that were below the limit of detection were recorded as 0. One-way ANOVA, Tukey's test where *P* ≤ .05, data shown as mean ± SEM with n = 3 biological replicates per group. Significant differences between groups are indicated as * = *P* ≤ .05. (C) *Hsd17b7* mRNA transcript levels in adult testes of wild-type (*Hsd17b3*^+/+^), heterozygous (*Hsd17b3*^+/−^) or homozygous (*Hsd17b3*^−/−^) mice. One-way ANOVA, Tukey's test where *P* ≤ .05, data shown as mean ± SEM with n = 5-8 biological replicates per group. Significant differences between groups are indicated as * = *P* ≤ .05, **** = *P* ≤ .0001.

The above results suggest that other enzyme(s) contribute to testosterone synthesis in the adult testis of mice lacking HSD17B3. We therefore investigated other hydroxysteroid dehydrogenase family proteins in the testes of *Hsd17b3* KO mice by LC-MS. HSD3B1, HSD3B6, HSD17B4, HSD17B7, HSD17B8, HSD17B10, and hydroxysteroid dehydrogenase-like 2 (HSDL2) were detected and the protein abundance between WT and *Hsd17b3* KO testes was compared (Fig. 7A). HSD17B7 was detected at low levels in 2 of the 3 WT samples and was undetectable in a third (Fig. 7A) but showed a ∼20-fold increase in *Hsd17b3* KO testes (Fig. 7A and 7B).

We then interrogated testicular *Hsd17b7* mRNA expression and showed that it was significantly increased in adult *Hsd17b3* KO mice (Fig. 7C), as previously observed (7). These results suggest that increased *Hsd17b7* mRNA and protein in the testis is a feature of *Hsd17b3* deficiency.

Next, we investigated the ability of HSD17B7 to convert androstenedione to testosterone. Two earlier studies suggested it does not utilize androstenedione as a substrate under different experimental conditions (32, 33); however, we sought to re-assess these observations using LC-MS to measure testosterone production.

**Figure 8.**
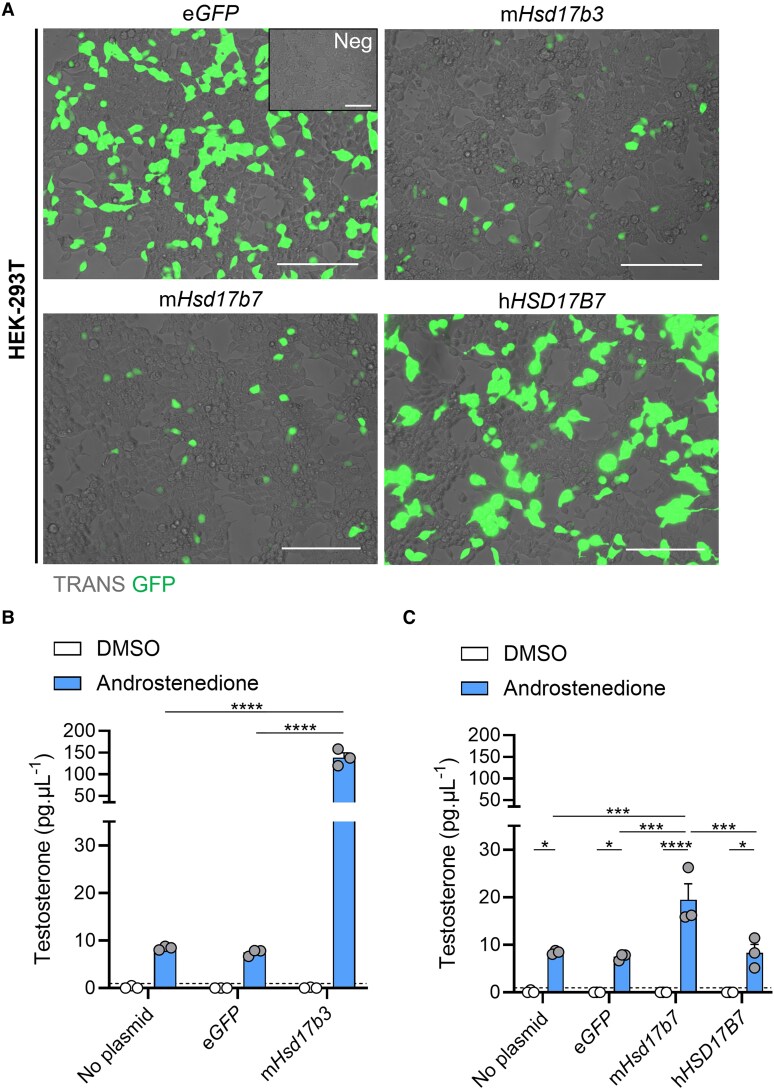
Mouse HSD17B7 can synthesize testosterone from androstenedione whereas human HSD17B7 cannot. (A) Representative images of HEK-293T cells transfected with either no plasmid (Neg), eGFP alone, or plasmids containing mouse (m) *Hsd17b3*, mouse m*Hsd17b7*, or human (h)*HSD17B7* with an e*GFP* reporter. Transfected cells indicated by eGFP expression (green). Scale bar: 150 μm. TRANS: transillumination. (B, C) Testosterone levels in culture media post transfection and following 24 hours of DMSO (vehicle, open bars) or 150 ng/mL androstenedione treatment (blue bars). Testosterone limit of detection = 0.98 pg/mL and is denoted by the dotted black line on the *y*-axis. Technical triplicates were averaged and plotted as biological replicates (n = 3). Two-way ANOVA, Tukey's test, data shown as mean ± SEM. Significant differences between groups are indicated as * = *P* ≤ .05, *** = *P* ≤ .001, **** = *P* ≤ .0001.

HEK-293T cells were transfected with plasmids expressing GFP reporters and steroidogenic enzymes, and 17β-hydroxysteroid dehydrogenase activity was assessed as the ability to convert androstenedione to testosterone. Successful transfection was confirmed by eGFP expression (Fig. 8A). Cells were treated with either DMSO (vehicle) or with androstenedione, and testosterone in the media was quantified. Cells transfected with mouse *Hsd17b3* were used as a positive control for testosterone synthesis, and a significant increase in testosterone production was observed in androstenedione-treated cells transfected with *Hsd17b3* compared to non-transfected or e*GFP* controls (Fig. 8B). Importantly, androstenedione-treated cells transfected with mouse *Hsd17b7* showed a significant increase in testosterone concentrations compared to androstenedione-treated, non-transfected and e*GFP* control cells (Fig. 8C). These data demonstrate that mouse HSD17B7 can synthesize testosterone from androstenedione under these conditions.

Finally, we examined the ability of human HSD17B7 to produce testosterone. Cells transfected with human *HSD17B7* had unchanged testosterone concentrations compared to non-transfected and e*GFP* controls and were significantly lower than the amounts produced by cells transfected with mouse *Hsd17b7* (Fig. 8C).

## Discussion

HSD17B3 has long been thought to be the canonical enzyme responsible for testicular testosterone production. In humans, loss-of-function mutations in *HSD17B3* cause disordered pre-pubertal testosterone production with consequent effects impairing reproductive organ development, with 46,XY individuals exhibiting impaired masculinization of external genitalia with internal male reproductive structures at birth (1, 3). However, mice with HSD17B3 deficiency exhibit normal male sexual development and fertility and continue to produce testosterone in the testis (4, 6, 7, 14), suggesting the existence of other enzymes that can produce testosterone in the absence of *Hsd17b3*.

HSD17B12 is a multifunctional enzyme which can utilize androstenedione as a substrate in mice to produce testosterone (18). We previously demonstrated that the HSD17B12 enzyme is expressed by the adult Leydig cells and there is a small but significant upregulation in testicular expression of *Hsd17b12* in *Hsd17b3* KO mice (6) and HSD17B12 has been hypothesized to contribute to testicular testosterone production in the absence of HSD17B3 (4, 6). Studies introducing mutations into residue 234 of the HSD17B12 enzyme showed that the presence of a phenylalanine amino acid blocks C19 steroids into the active site, preventing testosterone biosynthesis, however, smaller amino acids at position 234, such as alanine or leucine, enable testosterone biosynthesis (18, 20).

Transfection of HEK-293T cells confirmed that mouse HSD17B12 was able to produce testosterone in vitro but the human enzyme was not. Following substitution of the leucine amino acid at residue 234 in mouse HSD17B12 to a phenylalanine amino acid, as in human HSD17B12, we confirmed previous observations that the L234F substitution in mouse HSD17B12 reduced testosterone production to basal levels in HEK-293T cells (18, 20). Therefore, the L234F mutation in the mouse HSD17B12 enzyme provides an opportunity to inhibit HSD17B12's ability to use androstenedione as a substrate to produce testosterone but preserving expression of this enzyme throughout development.

We therefore used this mutation strategy to investigate whether HSD17B12 contributes to the maintenance of testicular testosterone production in the absence of HSD17B3 in mice. We generated a “humanized” L234F-mutated HSD17B12 mouse line and cross-bred this line with *Hsd17b3* KO mice. All male and female *Hsd17b3* KO mice expressing the mutated *Hsd17b12* developed the appropriate male and female reproductive tracts, respectively.

As *Hsd17b3* and *Hsd17b12* are expressed by the fetal testis during development (17, 34), the ablation of the androgenic function of these proteins in the context of continued normal development indicates the presence of one or more additional testosterone synthesizing enzymes during fetal development, with a recent publication indicating that a key contributor to this is HSD17B1 (17). Our observations are consistent with this previous study demonstrating that HSD17B1 is the predominant enzyme able to compensate for HSD17B3 deficiency during mouse fetal development (17). However, the testes of *Hsd17b1* + *Hsd17b3* double KO mice continue to produce lower but detectable levels of testosterone at birth (17). As HSD17B12 is expressed in the fetal testis (34), we propose that HSD17B12 activity could also contribute to the continued testosterone production observed in *Hsd17b1* +*Hsd17b3* double KO mice (17).

The alternate pathway of androgen biosynthesis can synthesize DHT independently of testosterone (14). Alternate pathway precursors including androsterone, 3α-diol and 3β-diol are increased in the circulation of adult *Hsd17b3* KO mice, but not in the testes (14). Similarly, DHT and alternate pathway precursors were unchanged in the testes of *Hsd17b3* KO mice expressing the mutated HSD17B12 enzyme compared to *Hsd17b3* KO mice expressing the WT HSD17B12, suggesting that the *Hsd17b12* mutation does not impact the ability of DHT production via the alternate pathway in the testes.

In adult *Hsd17b3* KO mice, introduction of the mutated HSD17B12 enzyme caused a small but significant decrease in testicular, but not circulating, testosterone and suggested that HSD17B12 contributes to ∼16% testicular testosterone synthesis in *Hsd17b3* KO mice. Significant decreases in seminal vesicle and testis weights were also observed, suggesting a small reduction in androgen bioactivity. However, testis and epididymal histology was unchanged with abundant sperm in the cauda epididymis, indicating that the loss of HSD17B12's ability to produce testosterone does not have a major impact on androgen bioactivity, sexual development, and adult reproductive function in male mice. Thus, we conclude that HSD17B12 does contribute to testicular testosterone production in mice, but it is not the sole enzyme responsible for the continued testicular testosterone production in *Hsd17b3* KO mice.

Therefore, the protein abundance of other 17-ketosteroid reductase enzymes in the testes of wild-type and *Hsd17b3* KO adult mice were investigated. HSD17B1 was not detected in the testes, aligning with previous studies demonstrating that HSD17B1 is down-regulated postnatally (35) and cannot compensate for HSD17B3 deficiency in adulthood (6, 17). HSD17B7 was deemed to be a strong candidate because both mRNA (7) and protein are significantly increased in *Hsd17b3* KO testes compared to wild-type, with the protein showing a particularly marked (∼20 fold) increase. HSD17B7 is a multifunctional enzyme that can utilize estrone, DHT and zymosterone as substrates (36-38). The mouse enzyme was first cloned in 1998 (32) and can convert the less active estrone to estradiol, a potent estrogen (32, 33). It also converts DHT into 3α-diol and, to a lesser extent, 3β-diol (33). However, previous studies suggested that the mouse enzyme is unable to convert androstenedione into testosterone (32, 33).

Our results revealed that, in transfected HEK-293T cells, mouse HSD17B7, but not human HSD17B7, can convert androstenedione to testosterone. Two earlier studies concluded that mouse HSD17B7 did not perform this conversion (32, 33); however, those studies employed reduced concentrations of substrate and shorter incubation times, which may have influenced the observed outcomes. Extrapolating from our findings, we suggest that HSD17B7 is unlikely to contribute to androgen bioactivity in 46,XY individuals with HSD17B3 deficiency (1, 3-5) but the mouse enzyme may be able to contribute to testosterone biosynthesis in *Hsd17b3* KO mice (6, 7, 17) in both fetal and postnatal life (34, 39). The KO of *Hsd17b7* in mice is embryonically lethal due to a defect in de novo cholesterol biosynthesis (36, 40). Because HSD17B7 is a multifunctional enzyme with various roles in steroidogenesis (36-38), future studies on its ability to contribute to testosterone biosynthesis in *Hsd17b3* KO mice would first require the identification of the specific amino acid residues responsible for the conversion of androstenedione to testosterone, as has been defined for HSD17B12 (18). Once such residues are defined, site-directed mutations and CRISPR/Cas9 technology could be used to produce mice carrying mutated *Hsd17b7* that is unable to produce testosterone. Introduction of the mutated *Hsd17b7* into *Hsd17b3* KO mice homozygous for *Hsd17b12^L234F^* could then be used to determine the contribution of HSD17B7 to testosterone synthesis.

We conclude that in the absence of the testosterone-biosynthetic enzyme HSD17B3, mice continue to produce basal testicular testosterone (6, 7), facilitated through the activity of other 17-ketosteroid reductase enzymes which contribute to the maintenance of testosterone biosynthesis. Our results show that mouse HSD17B12 can convert androstenedione to testosterone and, in mice lacking HSD17B3 function, HSD17B12 contributes to ∼16% of testicular testosterone production. However, testosterone production continues in mice lacking androgenic functions of both HSD17B3 and HSD17B12, suggesting that one or more further 17-ketosteroid reductase enzyme also contributes. Our results also show a marked increase in the testicular expression of HSD17B7 in the absence of *Hsd17b3*, and demonstrate that HSD17B7 can convert androstenedione to testosterone in vitro, suggesting that HSD17B7 may contribute to testosterone production in HSD17B3-deficient mice. The identification of 2 additional hydroxysteroid dehydrogenase enzymes that can synthesize testosterone in mice, but not humans, contributes toward a clearer understanding of the phenotypic differences between mouse and human HSD17B3 deficiency, where sexual development and fertility is preserved in mice, but disordered sexual development is seen in humans. These results suggest that the existence of multiple hydroxysteroid dehydrogenase enzymes capable of testosterone synthesis in mice is a safeguard for male sexual development and fertility in this species. These findings are consistent with the hypothesis that mice have increased plasticity in steroidogenic enzymes and multiple redundancies within steroidogenesis pathways. However, human orthologs have lost this plasticity, making humans more vulnerable to disordered sexual development following the loss of HSD17B3 function.

## Data Availability

Original data generated and analyzed during this study are included in this published article or in data repositories. The mass spectrometry proteomics data have been deposited to the ProteomeXchange Consortium via the PRIDE partner repository (41) with the dataset identifier PXD050748 and 10.6019/PXD050748.

## Abbreviations

3α-diol: 5α-androstane-3α,17β-diol
3β-diol: 5α-androstane-3β,17β-diol
AGD: anogenital distance
DHT: dihydrotestosterone
DMSO: dimethylsulfoxide
eGFP: enhanced green fluorescent protein
gDNA: genomic DNA
hCG: human chorionic gonadotropin
HSD17B12: 17β-hydroxysteroid dehydrogenase type 12
HSD17B3: 17β-hydroxysteroid dehydrogenase type 3
KO: knockout
PCR: polymerase chain reaction
qRT-PCR: quantitative reverse transcriptase polymerase chain reaction
WT: wild-type

## References

[1] Geissler WM, Davis DL, Wu L, et al Male pseudohermaphroditism caused by mutations of testicular 17β–hydroxysteroid dehydrogenase 3. Nat Genet. 1994;7(1):34‐39. 8075637 10.1038/ng0594-34

[2] O'Hara L, Smith LB. Androgen receptor roles in spermatogenesis and infertility. Best Pract Res Clin Endocrinol Metab. 2015;29(4):595‐605. 26303086 10.1016/j.beem.2015.04.006

[3] Gonçalves CI, Carriço J, Bastos M, Lemos MC. Disorder of sex development due to 17-beta-hydroxysteroid dehydrogenase type 3 deficiency: a case report and review of 70 different HSD17B3 mutations reported in 239 patients. Int J Mol Sci. 2022;23(17):10026. 36077423 10.3390/ijms231710026 PMC9456484

[4] Lawrence BM, O’Donnell L, Smith LB, Rebourcet D. New insights into testosterone biosynthesis: novel observations from HSD17B3 deficient mice. Int J Mol Sci. 2022;23(24):15555. 36555196 10.3390/ijms232415555 PMC9779265

[5] Mendonca BB, Gomes NL, Costa EM, et al 46,XY disorder of sex development (DSD) due to 17β-hydroxysteroid dehydrogenase type 3 deficiency. J Steroid Biochem Mol Biol. 2017;165(Pt A):79‐85. 27163392 10.1016/j.jsbmb.2016.05.002

[6] Rebourcet D, Mackay R, Darbey A, et al Ablation of the canonical testosterone production pathway via knockout of the steroidogenic enzyme HSD17B3, reveals a novel mechanism of testicular testosterone production. FASEB J. 2020;34(8):10373‐10386. 32557858 10.1096/fj.202000361R PMC7496839

[7] Sipilä P, Junnila A, Hakkarainen J, et al The lack of HSD17B3 in male mice results in disturbed Leydig cell maturation and endocrine imbalance akin to humans with HSD17B3 deficiency. FASEB J. 2020;34(5):6111‐6128. 32190925 10.1096/fj.201902384R

[8] Naamneh Elzenaty R, du Toit T, Flück CE. Basics of androgen synthesis and action. Best Pract Res Clin Endocrinol Metab. 2022;36(4):101665. 35595638 10.1016/j.beem.2022.101665

[9] van Weerden WM, Bierings HG, van Steenbrugge GJ, de Jong FH, Schröder FH. Adrenal glands of mouse and rat do not synthesize androgens. Life Sci. 1992;50(12):857‐861. 1312193 10.1016/0024-3205(92)90204-3

[10] Gannon AL, O'Hara L, Mason JI, et al Androgen receptor signalling in the male adrenal facilitates X-zone regression, cell turnover and protects against adrenal degeneration during ageing. Sci Rep. 2019;9(1):10457. 31320667 10.1038/s41598-019-46049-3 PMC6639311

[11] Huhtaniemi R, Oksala R, Knuuttila M, et al Adrenals contribute to growth of castration-resistant VCaP prostate cancer Xenografts. Am J Pathol. 2018;188(12):2890‐2901. 30273606 10.1016/j.ajpath.2018.07.029

[12] Adeniji AO, Chen M, Penning TM. AKR1C3 as a target in castrate resistant prostate cancer. J Steroid Biochem Mol Biol. 2013;137:136‐149. 23748150 10.1016/j.jsbmb.2013.05.012 PMC3805777

[13] Byrns MC, Mindnich R, Duan L, Penning TM. Overexpression of aldo-keto reductase 1C3 (AKR1C3) in LNCaP cells diverts androgen metabolism towards testosterone resulting in resistance to the 5α-reductase inhibitor finasteride. J Steroid Biochem Mol Biol. 2012;130(1-2):7‐15. 22265960 10.1016/j.jsbmb.2011.12.012 PMC3319280

[14] Lawrence BM, O'Donnell L, Gannon AL, et al Compensatory mechanisms that maintain androgen production in mice lacking key androgen biosynthetic enzymes. FASEB J. 2024;38(22):e70177. 39556387 10.1096/fj.202402093R PMC11698012

[15] Sha J, Baker P, O'Shaughnessy PJ. Both reductive forms of 17 beta-hydroxysteroid dehydrogenase (types 1 and 3) are expressed during development in the mouse testis. Biochem Biophys Res Commun. 1996;222(1):90‐94. 8630080 10.1006/bbrc.1996.0702

[16] Whiley PAF, O'Donnell L, Moody SC, et al Activin A determines steroid levels and composition in the fetal testis. Endocrinology. 2020;161(7):bqaa058. 32274496 10.1210/endocr/bqaa058

[17] Junnila A, Zhang FP, Martínez Nieto G, et al HSD17B1 compensates for HSD17B3 deficiency in fetal mouse testis but not in adults. Endocrinology. 2024;165(6):bqae056. 38785348 10.1210/endocr/bqae056

[18] Blanchard PG, Luu-The V. Differential androgen and estrogen substrates specificity in the mouse and primates type 12 17beta-hydroxysteroid dehydrogenase. J Endocrinol. 2007;194(2):449‐455. 17641292 10.1677/JOE-07-0144

[19] Suzuki H, Ozaki Y, Ijiri S, Gen K, Kazeto Y. 17β-Hydroxysteroid dehydrogenase type 12a responsible for testicular 11-ketotestosterone synthesis in the Japanese eel, Anguilla japonica. J Steroid Biochem Mol Biol. 2020;198:105550. 31778803 10.1016/j.jsbmb.2019.105550

[20] Luu-The V, Tremblay P, Labrie F. Characterization of type 12 17beta-hydroxysteroid dehydrogenase, an isoform of type 3 17beta-hydroxysteroid dehydrogenase responsible for estradiol formation in women. Mol Endocrinol. 2006;20(2):437‐443. 16166196 10.1210/me.2005-0058

[21] Bellemare V, Phaneuf D, Luu-The V. Target deletion of the bifunctional type 12 17β-hydroxysteroid dehydrogenase in mice results in reduction of androgen and estrogen levels in heterozygotes and embryonic lethality in homozygotes. Horm Mol Biol Clin Investig. 2010;2(3):311‐318. 10.1515/HMBCI.2010.036 25961203

[22] Mazza C, Breton R, Housset D, Fontecilla-Camps JC. Unusual charge stabilization of NADP+in 17beta-hydroxysteroid dehydrogenase. J Biol Chem. 1998;273(14):8145‐8152. 9525918 10.1074/jbc.273.14.8145

[23] Rantakari P, Lagerbohm H, Kaimainen M, et al Hydroxysteroid (17{beta}) dehydrogenase 12 is essential for mouse organogenesis and embryonic survival. Endocrinology. 2010;151(4):1893‐1901. 20130115 10.1210/en.2009-0929

[24] Saloniemi T, Jokela H, Strauss L, Pakarinen P, Poutanen M. The diversity of sex steroid action: novel functions of hydroxysteroid (17beta) dehydrogenases as revealed by genetically modified mouse models. J Endocrinol. 2012;212(1):27‐40. 22045753 10.1530/JOE-11-0315

[25] Skerrett-Byrne DA, Trigg NA, Bromfield EG, et al Proteomic dissection of the impact of environmental exposures on mouse seminal vesicle function. Mol Cell Proteomics. 2021;20:100107. 34089863 10.1016/j.mcpro.2021.100107 PMC8250459

[26] Skerrett-Byrne DA, Bromfield EG, Murray HC, et al Time-resolved proteomic profiling of cigarette smoke-induced experimental chronic obstructive pulmonary disease. Respirology. 2021;26(10):960‐973. 34224176 10.1111/resp.14111

[27] Skerrett-Byrne DA, Anderson AL, Hulse L, et al Proteomic analysis of koala (Phascolarctos cinereus) spermatozoa and prostatic bodies. Proteomics. 2021;21(19):e2100067. 34411425 10.1002/pmic.202100067

[28] Skerrett-Byrne DA, Anderson AL, Bromfield EG, et al Global profiling of the proteomic changes associated with the post-testicular maturation of mouse spermatozoa. Cell Rep. 2022;41(7):111655. 36384108 10.1016/j.celrep.2022.111655

[29] Smyth SP, Nixon B, Anderson AL, et al Elucidation of the protein composition of mouse seminal vesicle fluid. Proteomics. 2022;22(9):e2100227. 35014747 10.1002/pmic.202100227

[30] Mitchell RT, Mungall W, McKinnell C, et al Anogenital distance plasticity in adulthood: implications for its use as a biomarker of fetal androgen action. Endocrinology. 2015;156(1):24‐31. 25375036 10.1210/en.2014-1534 PMC4272396

[31] Mahendroo MS, Cala KM, Hess DL, Russell DW. Unexpected virilization in male mice lacking steroid 5 alpha-reductase enzymes. Endocrinology. 2001;142(11):4652‐4662. 11606430 10.1210/endo.142.11.8510 PMC4446976

[32] Nokelainen P, Peltoketo H, Vihko R, Vihko P. Expression cloning of a novel estrogenic mouse 17 beta-hydroxysteroid dehydrogenase/17-ketosteroid reductase (m17HSD7), previously described as a prolactin receptor-associated protein (PRAP) in rat. Mol Endocrinol. 1998;12(7):1048‐1059. 9658408 10.1210/mend.12.7.0134

[33] Torn S, Nokelainen P, Kurkela R, et al Production, purification, and functional analysis of recombinant human and mouse 17beta-hydroxysteroid dehydrogenase type 7. Biochem Biophys Res Commun. 2003;305(1):37‐45. 12732193 10.1016/s0006-291x(03)00694-6

[34] Tan K, Song HW, Wilkinson MF. Single-cell RNAseq analysis of testicular germ and somatic cell development during the perinatal period. Development. 2020;147(3):dev183251. 31964773 10.1242/dev.183251 PMC7033731

[35] O'Shaughnessy PJ, Baker PJ, Heikkila M, Vainio S, McMahon AP. Localization of 17beta-hydroxysteroid dehydrogenase/17-ketosteroid reductase isoform expression in the developing mouse testis–androstenedione is the major androgen secreted by fetal/neonatal leydig cells. Endocrinology. 2000;141(7):2631‐2637. 10875268 10.1210/endo.141.7.7545

[36] Jokela H, Rantakari P, Lamminen T, et al Hydroxysteroid (17beta) dehydrogenase 7 activity is essential for fetal de novo cholesterol synthesis and for neuroectodermal survival and cardiovascular differentiation in early mouse embryos. Endocrinology. 2010;151(4):1884‐1892. 20185768 10.1210/en.2009-0928

[37] Liu H, Robert A, Luu-The V. Cloning and characterization of human form 2 type 7 17beta-hydroxysteroid dehydrogenase, a primarily 3beta-keto reductase and estrogen activating and androgen inactivating enzyme. J Steroid Biochem Mol Biol. 2005;94(1-3):173‐179. 15862963 10.1016/j.jsbmb.2005.01.023

[38] Marijanovic Z, Laubner D, Moller G, et al Closing the gap: identification of human 3-ketosteroid reductase, the last unknown enzyme of mammalian cholesterol biosynthesis. Mol Endocrinol. 2003;17(9):1715‐1725. 12829805 10.1210/me.2002-0436

[39] Jameson SA, Natarajan A, Cool J, et al Temporal transcriptional profiling of somatic and germ cells reveals biased lineage priming of sexual fate in the fetal mouse gonad. PLoS Genet. 2012;8(3):e1002575. 22438826 10.1371/journal.pgen.1002575 PMC3305395

[40] Shehu A, Mao J, Gibori GB, et al Prolactin receptor-associated protein/17beta-hydroxysteroid dehydrogenase type 7 gene (Hsd17b7) plays a crucial role in embryonic development and fetal survival. Mol Endocrinol. 2008;22(10):2268‐2277. 18669642 10.1210/me.2008-0165 PMC2582539

[41] Perez-Riverol Y, Bai J, Bandla C, et al The PRIDE database resources in 2022: a hub for mass spectrometry-based proteomics evidences. Nucleic Acids Res. 2022;50(D1):D543‐d552. 34723319 10.1093/nar/gkab1038 PMC8728295

